# Veterinary antimicrobial card game improves antimicrobial selection skills in veterinary students

**DOI:** 10.3389/fvets.2025.1631567

**Published:** 2025-07-25

**Authors:** Jacob Wolf, Ashton C. Berger, Elayne P. Colon

**Affiliations:** ^1^Small Animal Clinical Sciences, College of Veterinary Medicine, University of Florida, Gainesville, FL, United States; ^2^School of Special Education, School Psychology, and Early Childhood Studies, College of Education, University of Florida, Gainesville, FL, United States

**Keywords:** serious games, educational games, antimicrobials, infectious disease, game-based learning, antibiotics

## Abstract

**Introduction:**

The inclusion of active learning in veterinary medical education has received significant focus in recent years. Game-based learning is an unconventional form of active learning and its use in education within the health sciences has been investigated.

**Methods:**

This study evaluated the use of a card game to teach antimicrobial use in dogs and cats with bacterial diseases as a supplement to traditional methods of teaching.

**Results:**

This study found that third- and fourth-year veterinary students’ comfort in antimicrobial decision-making increased for four infectious diseases following the inclusion of a card game during rounds. No similar improvement was noted for a disease that was not included in the card game. Students perceived that the game improved their understanding of antimicrobials in dogs and cats and students universally enjoyed playing the game.

**Discussion:**

The role of games in veterinary medical education should be further explored, especially studies that evaluate knowledge retention throughout the duration of their educational experience.

## Introduction

Active learning is a learner-centered form of education in which the learner is given the primary responsibility for constructing their own learning experience. Types of active learning that are commonly included in medical education include problem-based learning, team-based learning, flipped classrooms, peer-to-peer learning, patient simulations, virtual reality, audience response systems, and experiential learning (1). In medical education, active learning has been shown to improve critical thinking, knowledge, interpersonal interactions, and problem-solving abilities; learners also usually report a high degree of enjoyment of these activities. Active learning is thought to enhance higher-order thinking, rather than strictly recall memory (1, 2).

The role of serious gaming has gained considerable attention recently as a form of active learning within medical education. Serious games are most formally defined as “games [that] have an explicit and carefully thought-out educational purpose and are not intended to be played primarily for amusement” (3, 4). Serious games should exist as full-fledged games for a purpose other than pure entertainment. This differs from the term gamification, in which components or characteristics of games are applied to a pre-existing process (5). Game-based learning may be used to include both serious games and gamification. Types of serious games include card games, board games, escape rooms, and video games; gamification may include simulations and quizzes (5, 6). Other classification schemes organize serious games into virtual simulations, puzzles, quizzes, management simulations, platform games, board games, adaptation games, and adventure games (6).

The design and use of games for medical education may be particularly important in the modern era. Millennials and Generation Z read less than previous generations and are more familiar with image-immersive environments (7). During serious game design, creators should ensure the game has five major elements: a feeling of safety, clear, achievable, and meaningful goals, interactivity with intrinsic, formative feedback, the feeling of growth, and a design that occupies the senses (3). Additional considerations for serious game design include rapid gameplay, unpredictable elements, the presence of conflicts or challenges, the ability of the player to impact gameplay, interaction amongst participants, and rules intrinsic to gameplay (8, 9). Other game elements and essential components of gameplay have also been described (10). The choice of type of serious game design and the components of the game design should be tailored to the expected audience. Some studies have shown that women prefer puzzle games and games that highlight personal challenges, while men prefer competitive games (7). However, a recent study of game-based learning for dental students found greater score improvement with the use of collaborative games compared to competitive games (11). The desires of the educators must also be kept in mind when designing games for learning; a survey of health science educators found that they often prefer shorter, less complex games when teaching students (12).

Numerous postulated benefits to medical game-based learning have been proposed. Primarily, learners are allowed to solve clinical problems and make clinical decisions in a risk-free environment. Through game-based learning, learners also enhance collaboration and communication, think strategically, augment higher level learning such as analysis, and allow the learner to have fun. Game-based learning allows for immediate feedback, offers a self-paced, learner-controlled environment, provides intrinsic motivation, and is designed for repetition (3–6, 8, 9). Beyond the pedagogical impact, many studies have demonstrated that playing even traditional games, such as chess, may lead to improved mental health (13).

There is limited evidence for the use of card games to teach in veterinary medicine, though two recent studies demonstrated improved knowledge following the inclusion of card games in veterinary curricula (14, 15). The primary objective of this study was to evaluate the perceived effectiveness of a card game to teach antimicrobial prescriptions in dogs and cats to third- and fourth-year veterinary students. The topic of antimicrobial prescriptions was chosen due to the prevalence of infections treated in the emergency room, the degree of multidrug resistance observed there, and studies in human and veterinary medicine regarding compliance with published guidelines (16, 17).

## Methods

This study received an exemption from the University of Florida Institutional Review Board based on the research criteria (Protocol #17488). Students were included in the study during their 2-week emergency and critical care rotation. Verbal informed consent was obtained upon inclusion. The study was conducted by a veterinary critical care specialist on third- and fourth-year veterinary students. The study was conducted during their normal rounds time on the rotation, and groups typically included 4–7 students at a time. Veterinary students had already completed their pre-clinical courses, including antimicrobial pharmacology, prior to enrollment. Participation was voluntary; students could decline to be included in the research component of rounds. All data collection was de-identified.

### Pre-survey

Students were administered a written pre-survey (Supplementary material S1), which included demographic questions, questions about their experience with games, and questions about their comfort level with infectious diseases and antimicrobials using a 5-point Likert scale. It then asked students to rate their comfort using a 5-point Likert scale (1 the lowest and 5 the highest) in prescribing correct antimicrobials for five disease processes: pyometra, prostatitis, *Nocardia-*associated diseases, Lyme disease, and *Actinomyces-*associated diseases. Students were then asked to write what they believed to be the optimal drug to treat the disease underneath the disease.

### Traditional rounds

Following this, students received a traditional, verbal rounds session on antimicrobials approximately 30–45 min in length; all sessions were conducted by the same individual, a veterinary critical care specialist. No visual aids were used. Each rounds section proceeded in the same manner. Antimicrobials were discussed by mechanism of action (cell wall inhibitors, cell membrane inhibitors, protein synthesis inhibitors, and RNA/DNA synthesis inhibitors). Representative antimicrobials for each group were discussed, including their spectrum of activity, tissue penetration, and side effects; all infectious diseases on the surveys were discussed. Basic mechanisms of antibiotic resistance and the importance of responsible antibiotic use were also discussed. Students were then administered a written midpoint survey (Supplementary material S2) that asked the same questions regarding their comfort level with infectious disease and antimicrobials and their comfort in prescribing antimicrobials for the same five disease processes.

### Antimicrobial card game

Students immediately thereafter played an antimicrobial card game^1^ in which they were asked to pair antimicrobials with various infectious diseases in small animals. This section lasted approximately another 30–45 min. The game was created by a veterinary critical care specialist and human pediatrician and the cards were reviewed to validate content by numerous specialists, including other critical care specialists, an internist, a pharmacist, and a microbiologist. Prostatitis, Lyme disease, pyometra, and *Nocardia* were cards in the game; no *Actinomyces* was created in order to create a control to monitor the impact of the card game. In short, each student had 5 antimicrobial cards in their hand. In front of them were 5 disease cards with short case descriptions; each card had a different number of points listed based on the difficulty of treating the disease. On their turn, students attempted to treat one of the diseases; they played a card and individually looked at the back of the card which contained the correct answer(s) for treatment. If they were correct, they received the points on the card, discarded their antimicrobial card, received a new antimicrobial card, and a new disease card was placed onto the table. Play then continued to the next person. If they were incorrect, they placed the disease card back down, retrieved their antimicrobial card, and play passed to the next person. The facilitator occasionally provided clarification or prompted discussion regarding difficult or controversial prescribing practices once the correct answer had been established.

### Post-survey

Following completion of the game, students were administered a written post-survey (Supplementary material S3) that asked the same questions regarding their comfort level with infectious disease and antimicrobials and their comfort in prescribing antimicrobials for the same five disease processes, as well as questions about the gameplay itself. Correct antimicrobial answers were pre-determined by the experts listed above.

### Data analysis

Student demographics and survey response data were aggregated and analyzed using the Python 3^2^ scripting language. Demographics were assessed to determine if they followed the Gaussian probability distribution. Comparisons between responses were made using the Wilcoxon signed rank test for agnosticism of the underlying distributions, but summary statistics were represented as means when comparing question responses between different timepoints for consistency. For evaluating student accuracy of antimicrobial selection, the proportion of correct responses over total available responses for each question was calculated at each timepoint. The changes in accuracy over time were then calculated as the percent change in the proportion of correct answers for a question between two consecutive timepoints.

## Results

A total of 50 students were included in the study. The median age was 25 years (range: 22–37 years); 4 students (8%) identified as male, and 46 students (92%) identified as female. Six students (12%) reported that they regularly play role playing games, 12 (24%) regularly play simulation-based games, 30 (60%) regularly play video games, 33 (66%) regularly play quiz games, 41 (82%) regularly play board games, and 44 (88%) regularly play card games. For those that participated in each game, the median amount of time spent each week playing that game type was estimated to be between 1.5–3.5 h. Student age and hours spent gaming per week did not follow the Gaussian probability distribution and therefore were described using median values for summary statistics.

**Figure 1:**
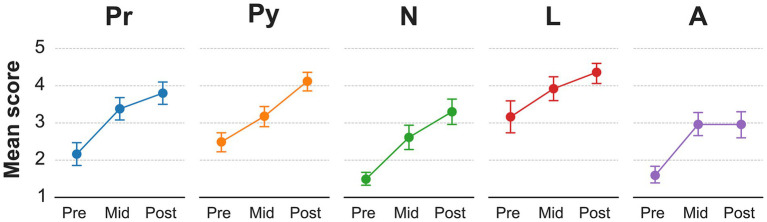
Veterinary student comfort in prescribing antimicrobials after traditional rounds and a card 448 game. Mean score for self-evaluated veterinary student comfort in prescribing antimicrobials for various disease processes prior to participation (pre), after traditional rounds (mid), and after an antimicrobial card game (post) (Likert scale 1–5, with 1 least confident and 5 most confident). Pr, prostatitis; Py, pyometra; N, *Nocardia*; L, Lyme disease; A, *Actinomyces.*

The mean answers student comfort level with antimicrobials and infectious diseases for each time point can be found in Table 1. Student responses for each question were evaluated separately at the pre-, mid-, and post-survey timepoints; the responses for most questions at each timepoint followed the Gaussian distribution. When survey responses for all questions were aggregated by timepoint, the response distributions became more skewed and deviated from the Gaussian. Scores across each question increased significantly after the traditional rounds session and increased further after the card game. Students indicated that following both the traditional rounds and the card game they had greater understanding of the mechanism of action of antibiotics, of bacterial infections in dogs and cats, and of antibiotic prescribing practices. The mean comfort level for individual disease processes at each time point can be found in Table 2. Students’ comfort in prescribing antimicrobials for all five representative diseases improved significantly following traditional rounds. Students’ comfort in prescribing antimicrobials further improved for four representative diseases following the card game (Figure 1). There was no change in comfort for antimicrobial prescribing after the card game for *Actinomyces,* which was the sole infectious disease not contained in the game (Figure 1).

**Table 1:** Student comfort level with infectious disease and antimicrobials changed over time.

Question	Pre-survey	Midpoint survey	Post survey	Difference between pre- and midpoint survey	Difference between midpoint and post-survey
I have a good understanding of bacterial infections in dogs and cats	3.0 ± 0.67	3.3 ± 0.87	3.6 ± 0.73	0.28 (*p* = 0.004)	0.28 (*p* = 0.001)
I have a good understanding of when to prescribe antibiotics for diseases in dogs and cats	3.4 ± 0.81	3.7 ± 0.67	4.0 ± 0.73	0.3 (*p* = 0.008)	0.24 (*p* = 0.007)
I have a good understanding of which antibiotics to prescribe for specific conditions in dogs and cats	2.3 ± 0.82	3.2 ± 0.92	3.6 ± 0.80	0.92 (*p* < 0.0001)	0.4 (*p* < 0.001)
I understand the mechanism of action of most antibiotics	2.3 ± 0.88	3.3 ± 0.96	3.5 ± 0.99	1.06 (*p* < 0.0001)	0.2 (*p* = 0.033)
I understand the antibiotic susceptibility of many bacteria that affect dogs and cats	2.4 ± 0.92	3.2 ± 0.95	3.4 ± 0.99	0.76 (*p* < 0.0001)	0.22 (*p* = 0.022)
I find learning about antibiotics fun	3.3 ± 1.1	3.7 ± 1.0	4.1 ± 1.1	0.44 (*p* < 0.001)	0.4 (*p* < 0.001)
I find learning about bacteria fun	3.3 ± 1.2	3.7 ± 1.1	4.0 ± 1.1	0.32 (*p* = 0.004)	0.36 (*p* < 0.001)

Values are presented as means. *p*-values were calculated using the Wilcoxon signed rank test. Students were asked to rate their comfort level/agreement from 1 to 5 with 1 as the least comfortable and 5 as the most comfortable. ± indicates one standard deviation.

**Table 2:** Student comfort level with antimicrobial prescriptions by individual disease type.

Question	Pre-survey	Midpoint survey	Post survey	Difference between pre- and midpoint survey	Difference between midpoint and post-survey
Prostatitis	2.2 ± 1.1	3.4 ± 1.1	3.8 ± 1.1	1.18 (*p* < 0.00001)	0.43 (*p* < 0.0001)
Pyometra	2.5 ± 1.0	3.2 ± 1.0	4.1 ± 0.94	0.65 (*p* < 0.001)	0.98 (*p* < 0.00001)
*Nocardia*	1.5 ± 0.58	2.6 ± 1.2	3.3 ± 1.2	1.08 (*p* < 0.00001)	0.67 (*p* < 0.001)
Lyme disease	3.2 ± 1.5	3.9 ± 1.2	4.4 ± 0.94	0.73 (*p* < 0.0001)	0.5 (*p* = 0.009)
*Actinomyces*	1.6 ± 0.81	3.0 ± 1.2	3.0 ± 1.3	1.35 (*p* < 0.00001)	0.00 (*p* = 0.93)

Values are presented as means. *p*-values were calculated using the Wilcoxon signed rank test. Students were asked to rate their comfort level from 1 to 5 with 1 as the least comfortable and 5 as the most comfortable. ± indicates one standard deviation.

Few students provided written answers for the antimicrobial that they would prescribe for each individual disease process; the number of answers and the percent correct for each disease are displayed in Table 3. No students provided answers to the pre-survey for *Actinomyces* and *Nocardia* and, as such, percent change from the pre- to mid-survey timepoints could not be calculated. However, for those that responded, incorporation of the card game did increase the percentage of correct answers for many of the disease processes evaluated.

**Table 3:** Student antimicrobial prescriptions for individual infectious disease processes.

Question	Pre-survey	Midpoint survey	Post survey	Difference between pre- and midpoint survey	Difference between midpoint and post-survey
Prostatitis	4/4	15/15	18/18	*0%	*0%
Pyometra	4/11	8/15	18/21	+47%	+61%
*Nocardia*	0/0	4/7	10/14	–	+25%
Lyme disease	18/18	22/22	25/25	*0%	*0%
*Actinomyces*	0/0	4/8	5/7	–	+43%

Values are displayed as the number of correct answers over the number of students who wrote an answer. Differences are represented as the percent change in responders answering correctly between two different survey timepoints. * = Percent change could not be computed because 100% of responders were correct at all timepoints for prostatitis and Lyme disease. – = Percent change could not be computed due to a lack of responders at the pre-survey timepoint for Nocardia and Actinomyces.

One student did not answer questions on game structure. All remaining students (*n* = 49) found that the game possessed intrinsic motivation, provided feedback, allowed for decision-making, fostered a safe environment, and engaged the senses. All students (*n* = 49) felt they experienced growth and found the game fun. The majority of respondents found the game cognitively challenging (98%) and felt the game had elements of unpredictability (96%). All students said they would play the game again.

## Discussion

This study found that an antimicrobial card game used as a supplement to traditional rounds can increase student comfort in prescribing antimicrobials for infectious diseases included in the card game. A similar benefit was not found for an infectious disease not included in the card game. Additionally, this study found that students enjoyed playing the card game and would play it again; students found the study of bacteria and antimicrobial more enjoyable after gameplay. Finally, correct prescriptions for various infectious diseases increased following incorporation of the card game. These findings are in agreement with the growing body of literature that supports game-based learning in medical curricula.

A previous study evaluated clinical year veterinary students who were surveyed before and after playing a board game designed to teach swine antimicrobial therapy. Similar to the current study, following the class, student scores increased significantly, and students universally enjoyed the game. No control group was used in this study (14). Another study evaluated third-year veterinary student interpretations of urological radiographic abnormalities. This study randomized students to either a control group that studied traditional radiographic cases or a card game designed to teach radiographic abnormalities. The study found that students who played the card game scored higher on posttest evaluations than the control group; this higher score was also present 1 week later (15). Another study evaluated the use of a digital board game to teach histology to first-year veterinary students. That study found that incorporation of the board game resulted in higher student engagement, enjoyment, and motivation (18). The present study agrees with these studies that game-based learning can lead to acquisition of knowledge and provide a fun, safe environment for students, providing further evidence for the use of game-based learning in veterinary education. Indeed, some veterinary schools have created initiatives to further incorporate game-based learning into their veterinary curriculum (19).

Though not a direct corollary, simulators and virtual reality in veterinary medical education have been extensively studied. Systematic reviews of simulators in veterinary education have found that simulation-based training led to improved knowledge and skills compared to traditional teaching methods (20, 21). Virtual reality training has also shown promise in veterinary education, including in anesthesia and orthopedics, though further studies are needed to compare its effectiveness to traditional learning methods (22–24). A simulated clinical setting using Second Life was also evaluated as a method to teach clinical reasoning to first-year veterinary students. The study found that students and faculty believed that this simulation created an authentic learning environment and that students enjoyed its inclusion in the curriculum (25). Escape rooms have also been evaluated as a form of game-based learning in veterinary education. Generally, learners reported that escape rooms fostered collaboration and communication and that their knowledge base improved following their participation. In these scenarios, students were able to incorporate complex information, synthesize it, and apply it to a clinical scenario (26, 27). These studies further support the intentional incorporation of a variety of game-based learning into medical education; however, given their different structure from the game used in the current study, it is unclear how directly applicable these findings are. Clearly, the term “game-based learning” encompasses a broad swath of games; additional studies are needed to determine which games are most educational within veterinary medicine which games are most suited for various subjects or skills.

Similar findings have been documented in human medical education. One study found that the use of video games in radiology improved radiographic interpretation abilities, visual-motor coordination, and increased visual spatial resolution (3). Surgeons and surgery residents have demonstrated improved error rates and faster procedure times after playing video games, particularly for laparoscopic procedures (28, 29). Several randomized controlled trials have identified higher posttest scores in medical learners who participated in game-based learning when compared to conventional learning (5, 30). Individual board games in human medicine used to teach anatomy and public health have also shown improvements in student knowledge (31, 32). A card game used to supplement teaching of bacteriology found increased knowledge acquisition in medical students who used the game. Because of this, the game is now published by the French Society of Microbiology and provided to all French medical and pharmacy schools (33). Game-based learning may be best when used to supplement, rather than replace, conventional learning (5). The present study was designed with that in mind and is why we elected not to create two separate study groups (a traditional learning group only and a game-based learning group). The breadth of human literature supporting the use of game-based learning, taken together with the present study, indicates that this style of learning can be informative, interactive, and enjoyable for learners. Additional studies are needed to determine whether the information learned in serious games influences the behavior of future clinicians.

This study has several limitations. Few students provided answers for which antimicrobial they would prescribe for each disease scenario, likely since the survey did not prompt them to provide this in an obvious enough way. This precluded in-depth analysis of student knowledge, requiring the study to rely more heavily on student reactions. Future studies should attempt to evaluate knowledge and associated behavioral changes. As mentioned above, we elected not to have a control group in which students did not play the card game. While having a control and study group instead of evaluations after each intervention may have provided important information, we do not believe that would be consistent with how a game would be used within a veterinary curriculum. We also did not randomize whether students would receive traditional rounds or the card game first, as we believe a basic understanding of antimicrobials is necessary before use of the game. This study had significantly more female (92%) than female (8%) students, likely reflecting the demographic makeup of many veterinary schools in the United States. Therefore, this study did not include enough male students or students of various ages to evaluate whether these factors influence knowledge acquisition and game enjoyment. Lastly, this study did not evaluate student knowledge over time and whether game-based learning may have an impact on knowledge retention. Future studies that focus on retention, and game repetition, are needed. Certain diseases (*Actinomyces*, *Nocardia*) had much lower response rates than others (*Borrelia*); this is likely due to the prevalence of these diseases in the United States and the time of the curriculum devoted to their study. No students provided an empiric antibiotic for *Actinomyces* or *Nocardia* on the pre-survey; this was likely due to baseline lack of familiarity with these bacteria.

This study found that game-based learning improves student comfort when prescribing antimicrobials across artificial clinical scenarios and may increase knowledge acquisition. This is important, especially in the context of antimicrobials, given the rapid rise of antimicrobial resistance due to improper prescribing practices. These findings reinforce a growing body of literature that demonstrates that game-based learning should have a role within veterinary education. Future studies that focus on repetition of educational games and knowledge retention over time using game-based learning are needed to determine the best manner in which to implement game-based learning.

## Data Availability

The raw data supporting the conclusions of this article will be made available by the authors, without undue reservation.

## Footnotes

^1^Empiric Small Animal Veterinary Card Game, Empiric, Portland, OR.

^2^Python 3, Python Software Foundation, Beaverton, OR.
