# Supplementary material for: Veterinary antimicrobial card game improves antimicrobial selection skills in veterinary students
Source: Front Vet Sci. 2025 Jul 25;12:1631567. doi: 10.3389/fvets.2025.1631567 (PMC12332509; doi:10.3389/fvets.2025.1631567)
Supplement: Supplementary file 3 [file Data_Sheet_3.PDF]

Student ID #: _____________

Student Survey (Post)

1. For the following statements, answer 1 to 5 (with 5 being the strongest/most comfortable and 1 the weakest/least comfortable):

**I have a good understanding of bacterial infections in dogs and cats**	1	2	3	4	5
**I have a good understanding of when to prescribe antibiotics for diseases in dogs and cats**	1	2	3	4	5
**I have a good understanding of which antibiotics to prescribe for specific conditions in dogs and cats**	1	2	3	4	5
**I understand the mechanism of action of most antibiotics**	1	2	3	4	5
**I understand the antibiotic susceptibility of many bacteria that affect dogs and cats**	1	2	3	4	5
**I find learning about antibiotics fun**	1	2	3	4	5
**I find learning about bacteria fun**	1	2	3	4	5

2. For the following disease processes, list your comfort level deciding on proper antibiotic selection (with 5 being the most comfortable and 1 the least comfortable). If you know an optimal antibiotic to treat with, please write it underneath the disease process.

**Prostatitis**	1	2	3	4	5
**Pyometra**	1	2	3	4	5
**Nocardia**	1	2	3	4	5
**Lyme disease**	1	2	3	4	5
**Actinomyces**	1	2	3	4	5

Student ID #: _____________

3. Please indicate whether you found the game possessed the following attributes:

**Cognitively challenging**	Yes	No
**Elements of unpredictability**	Yes	No
**Intrinsic motivation**	Yes	No
**Provided feedback**	Yes	No
**Allowed for decision-making**	Yes	No
**Fun**	Yes	No
**Engaged the senses**	Yes	No
**Experienced growth**	Yes	No
**Fostered a safe environment**	Yes	No

4. Would you play this game again? Yes No

5. If you have any other comments about this process, please include them below:
